# Criteria to Measure Social Media Value in Health Care Settings: Narrative Literature Review

**DOI:** 10.2196/14684

**Published:** 2019-12-16

**Authors:** Chukwuma Ukoha, Andrew Stranieri

**Affiliations:** 1 Centre for Informatics and Applied Optimisation Federation University Australia Ballarat Australia

**Keywords:** social media, information systems, health care, value, measurement, criteria

## Abstract

**Background:**

With the growing use of social media in health care settings, there is a need to measure outcomes resulting from its use to ensure continuous performance improvement. Despite the need for measurement, a unified approach for measuring the value of social media used in health care remains elusive.

**Objective:**

This study aimed to elucidate how the value of social media in health care settings can be ascertained and to taxonomically identify steps and techniques in social media measurement from a review of relevant literature.

**Methods:**

A total of 65 relevant articles drawn from 341 articles on the subject of measuring social media in health care settings were qualitatively analyzed and synthesized. The articles were selected from the literature from diverse disciplines including business, information systems, medical informatics, and medicine.

**Results:**

The review of the literature showed different levels and focus of analysis when measuring the value of social media in health care settings. It equally showed that there are various metrics for measurement, levels of measurement, approaches to measurement, and scales of measurement. Each may be relevant, depending on the use case of social media in health care.

**Conclusions:**

A comprehensive yardstick is required to simplify the measurement of outcomes resulting from the use of social media in health care. At the moment, there is neither a consensus on what indicators to measure nor on how to measure them. We hope that this review is used as a starting point to create a comprehensive measurement criterion for social media used in health care.

## Introduction

### Background

The use of social media in health care settings is increasingly becoming prevalent [1,2]. Social media used in health care settings can broadly be grouped into 2 categories—general purpose Web-based social networks and online health communities [3]. General purpose Web-based social networks include most Web 2.0 websites and applications such as Facebook and Twitter that enable users to create and share content or to participate in social networking. Online health communities, on the contrary, are special purpose platforms such as PatientsLikeMe and MedHelp that provide a means for health care professionals, patients, and their families to share information about an illness, seek and offer support, and connect with others in similar circumstances.

Social media refers to internet-based applications that enable the creation and exchange of user-generated content [4]. It is a complex combination of sociology and technology [5]. Social media facilitates social interaction and allows the creation of virtual communities. In terms of technology, social media is underpinned by Web 2.0 technology that enables people to create, share, collaborate, and communicate. Use cases of social media in health care can broadly be categorized into 4 major types, namely professional to professional (P2P), professional to consumer (P2C), consumer to consumer (C2C), and consumer to professional (C2P) [3]. P2C represents a situation in which a health care professional provides support to a health consumer; C2P represents health consumers who provide support to health care professionals by way of contributing their experience and opinions; and P2P and C2C represent interactions and exchange of support among members of the respective Health 2.0 user groups.

Social media has become a tool of choice for members of the medical profession who see it as a medium to connect, engage, and influence their audience [6]. In many organizations, social media serves both as a medium to communicate with customers and as a medium to communicate internally [7]. Thus, it has become a medium for the health care community to network, develop their skills, and forge their identity [8]. Health care professionals leverage social media to build, reinforce, and maintain professional relationships with colleagues and to share information [9]. Health care providers use social media to promote their organizations, amplify word-of-mouth effects, build strong relationships with both existing and potential customers, improve brand awareness, and increase volume of traffic to website [10,11]. Patients can also access information relevant to their condition through social media [1]. Such medical information may include links to health resources or images or videos with relevant health content [12].

### Objectives

Research to date on the value of social media in health care settings has largely focused on the use and value of the application to health care providers [1,13-15] and patients [16-21]. Fewer studies have focused on measuring the value of social media in health care settings. As health care becomes more patient centered and outcome driven, stakeholders need to be able to report and measure outcomes arising from social media use [2,22,23]. In health care settings, there is considerable interest in exploring how best to ascertain value derived from social media initiatives [2,22-24]. However, measuring the value of social media is a conundrum [5,7,25,26]. The value of an information system (IS) is often linked with its ability to satisfy specific needs [27]. Some have argued that the value of social media has to do with its effectiveness as a marketing tool and suggest that metrics such as hit rate and follower numbers that measure the marketing reach be used to measure social media value [28,29]. However, in health care settings, the use of marketing-based metrics alone may not be sufficient to measure the value of social media, given that, in health care, ensuring better health outcomes for patients rather than attracting more patients is the paramount objective. Furthermore, the impact of social media on patient outcomes can be subtle; thus, it may not be measurable using traditional IS metrics alone [30].

Although relevant literature reveals a kaleidoscope of approaches that can potentially be used to measure the value of social media in health care settings, there is little consensus on what indicators to measure or on how to measure them [15,31]. To clarify this complex phenomenon, we explored different approaches to measure the value of social media in health care settings proposed in previous studies. We deployed a taxonomic approach to elucidate the measurement criteria and to present them in a way that illustrates common features. This allowed the classification of diverse measurement approaches according to a predetermined system, with the resulting catalog being used to provide a conceptual framework for discussion, analysis, and information retrieval. Taxonomies related to social media use have been identified to be critical to understanding the state of the art in research in this area [32].

Many measurement criteria of health care social media identified in the relatively few literatures on the subject are confusing and difficult to apply [33]. To date, however, there is no scholarly paper (known to the authors) that reviews the current knowledge, including substantive findings, and theoretical and methodological contributions in this area. This paper fills this gap. By reviewing and elucidating current measurement criteria propounded in the relevant literature, we hoped to contribute to a better understanding of the value of social media in health care.

The aim of this study was to elucidate how the value of social media in health care settings can be ascertained and to taxonomically identify the steps and techniques in social media measurement from a review of relevant literature.

## Methods

### Overview

To address the objective of this study, we sought to understand existing research [34] from a theoretical and contextual point of view [35]. Although it is not a requirement for narrative review articles to list the types of databases and methodological approaches used to conduct the review or the evaluation criteria for inclusion of retrieved articles [35], we provided this information to ensure methodological transparency.

**Figure 1:**
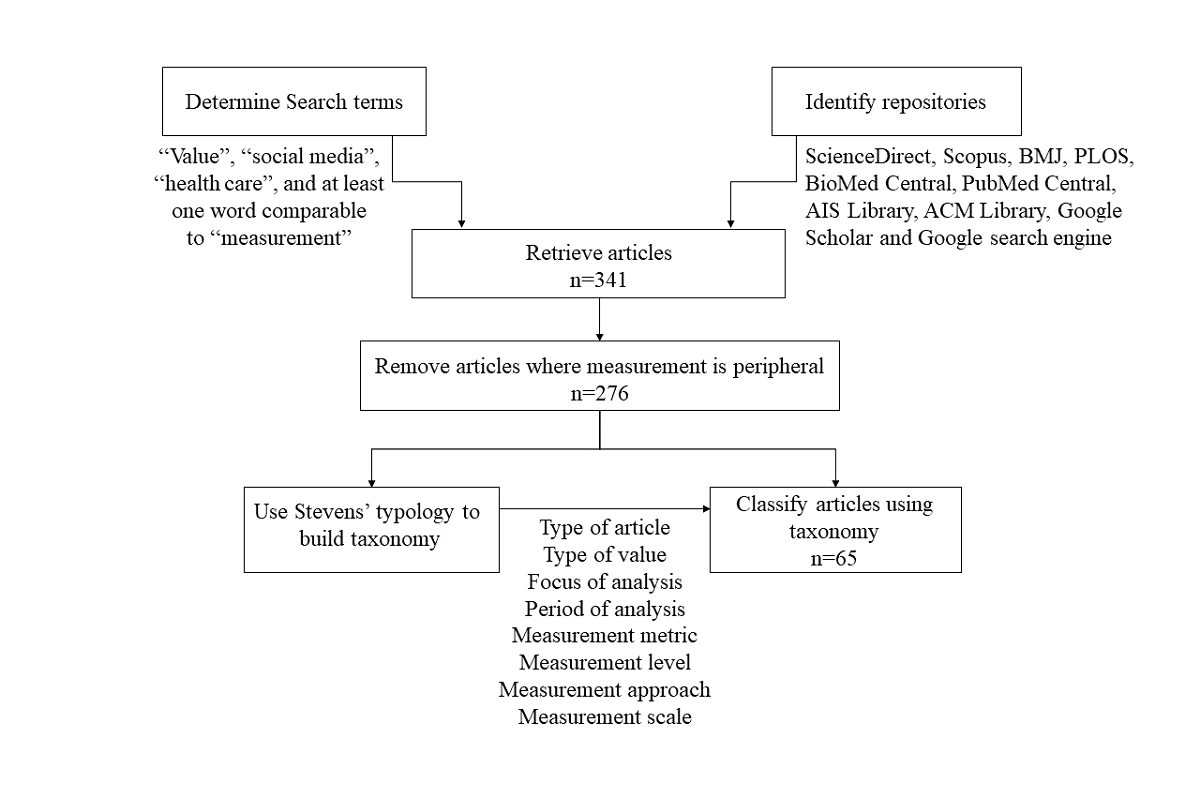
Sequence of activities in taxonomy development.

Repositories containing the full text of relevant research articles were first identified. Search terms described below were constructed and applied to retrieve articles from the repositories. Retrieved articles were culled so that only the articles that were specifically related to measuring value in health care, ISs, and social media deployments remained. We then performed an analysis of the retrieved articles guided by Stevens’ [36] measurement typologies also described in the following section. Figure 1 illustrates the activities performed to arrive at a taxonomy and apply the taxonomy to published studies related to determining value from social media use in health care settings.

### Search Criteria

We included articles in journals, conference proceedings, and reports published in English that specifically or broadly discussed measuring ISs value, measuring social media value, and measuring value in health care. Only papers published since 2009 on measuring the value of social media in health care settings were included. Given that the literature on social media use in health care settings is scattered across repositories, the search was not limited to specific databases. Rather, relevant literature was collected from a variety of online databases, that spanned IS, medical informatics, medical and business journals, and conference proceedings and reports [37]. Databases searched included ScienceDirect, Scopus, BMJ, BioMed Central, PubMed Central, PLOS, AIS Library, and ACM Library and Google Scholar as a general database. Furthermore, the Google search engine was used to identify other articles that might not have been accessible in the online databases. To identify published articles pertaining to measuring the value of social media in health care, academic databases were probed using multiple search terms. All searches included the following terms: “value,” “social media,” “health care” and at least one word comparable to “measurement.” This ensured that the review covered substantial relevant literature on the subject and was not confined to one research methodology, one set of journals, or one geographic region [38].

### Article Selection and Analysis

The titles and abstracts of 341 articles were reviewed. After sorting through the articles, papers merely discussing the definitions and types of social media, without suggesting the value or measurement criteria, were deemed not relevant to the review, and thus were excluded. At the end, only relevant publications (n=65) were included in the review.

The intent of reviewing the relevant literature on the subject was not to conduct a systematic review; rather, the intention was to enhance the quality of the arguments and assertions presented herein by reviewing a sufficient amount of the literature [39], given that suitable measurement criteria for social media have to be underpinned by relevant theory and embedded in the literature [40].

A narrative review of the published literature that describes measurement criteria for health care social media was conducted after selecting relevant articles. A narrative review approach ensured that the narrative thread would not be lost in the restrictive rules of a systematic review and that issues that require the wider scoping of a narrative review are addressed [41]. While qualitatively analyzing and synthesizing relevant literature, a conscious effort was made to avoid simply repeating ideas presented in the reviewed articles without elucidating how they apply to the subject matter. Thus, this study makes an academic contribution by synthesizing the available material and offering a scholarly analysis [34].

### Guideline for Analysis

The phenomenological nature of value and its various perspectives and viewpoints make it challenging to develop suitable measurement criteria for evaluation [42,43]. This study tries to make sense of the different approaches and to make relevant recommendations accordingly. As is usually the case with narrative review articles, this paper does not adopt methodological approaches that would answer specific quantitative questions [35]. Rather, it uses qualitative methods, using headings and subheadings, to discuss the phenomenon that is the focus of the paper being reviewed [35].

To have a consistent basis to compare and contrast the distinct yardsticks used in the measurement of health care social media, a taxonomic approach was adopted, using Stevens’ [36] measurement typologies as a guideline. Stevens introduced a theory of levels of measurement in 1946. Level of measurement or scale of measure is a classification that describes the nature of information within the values assigned to variables. According to Stevens, all measurements could be conducted using at least one of four different types of scales called *nominal*, *ordinal*, *interval*, and *ratio* [36]. His measurement scales allow for both qualitative and quantitative measurements and are widely used by researchers. In this study, we used the scales as a framework for taxonomizing the various measurement criteria for social media in health care. This taxonomy would enable a better understanding of how health care providers can measure their social media.

## Results

### Taxonomy and Criteria

A variety of approaches for measuring the value of social media in health care settings were identified in this review. We have argued that the choice of measurement yardstick depends on the context, that is, the objective that underpins a health care provider’s use of social media.

The following sections present the conclusions reached based on the literature reviewed. Table 1 illustrates the taxonomy derived from relevant literature. It describes the content and characteristics of relevant literature, including the objectives, focus of analysis, period of analysis, measure of value adopted, and the type of value identified. It also presents how Stevens’ [36] measurement typologies can facilitate a better understanding of the existing measurement criteria for health care social media.

**Table 1:** Taxonomy derived from the literature on measuring health care social media value.

Taxonomic class	Category A^a^	Category B^a^	Category C^a^	Category D^a^
Type of article	IS^b^ or IT^c^ Business Medical eHealth	Peer reviewed Non–peer reviewed	—^d^	—
Type of value	IS value Health value	Utilitarian value Hedonic value	Instrumental value Intrinsic value	Contextual value
Focus of analysis	Value of health care social media and the need for its measurement	Measurement of outcomes resulting from the use of health care social media	Deriving health information from measuring social media data	—
Period of analysis	Pre–social media adoption	Post–social media adoption	—	—
Measurement metric	Monetary value	Non–monetary value	—	—
Measurement level	Micro Aggregate	International National Industry Organizational Individual	—	—
Measurement approach	Quantitative^e^	Qualitative^f^	Mixed method^g^	—
Measurement scale	Nominal^h^	Ordinal^i^	Interval^j^	Ratio^k^

^a^Category, as used here, refers to alternatives within a taxonomic class, in no particular order.

^b^IS: information systems.

^c^IT: information technology.

^d^Some taxonomic classes have fewer categories, hence the empty cells.

^e^Quantitative measurement involves the use of an interval or a ratio scale.

^f^Qualitative measurement involves the use of a nominal or an ordinal scale.

^g^The mixed method approach involves the use of both quantitative and qualitative methods.

^h^A nominal scale is used for classification or grouping (eg, = or ≠).

^i^An ordinal scale is used for comparison and sorting (eg, > or <).

^j^An interval scale is used to determine difference or affinity (eg, + or −).

^k^A ratio scale is used to determine magnitude or amount (eg, x or /).

### Type of Article

The analysis of the literature shows that existing literature relevant to the value of social media in health care settings broadly fits into 2 categories: peer reviewed sources and non–peer reviewed sources. Peer reviewed sources outnumbered non–peer reviewed sources.

Furthermore, the literature on the subject can be categorized based on the discipline toward which the publication leans. Some of the relevant literature were published in IS publications, business publications, medical publications, and medical informatics publications, which discuss issues that intersect IS and health care. Most articles cited in this review are from medical informatics publications.

### Type of Value

The concept of *value* is multidimensional and its conceptualization depends on the perspective used [44]. When measuring the value of social media in health care, the dilemma is often what metric to use, whether to use metrics that measure IS value or health value, whether to use metrics that measure utilitarian or hedonic value, or whether to measure instrumental or intrinsic value, as outlined below. The type of value to measure depends on the context of use.

#### Information System Value and Health Value

IS researchers view value as the worth, desirability, or utility of IS artifacts [43], as happy endings in terms of system impact [45] or as the positive outcome that is created through user-system interaction [46]. In health care, however, value has been conceptualized as *the health outcomes achieved per dollar spent* [30,47,48] or as patient outcomes divided by total costs for providing care [49]. Value in health care is evidenced by the outcomes achieved [30]. However, some health care outcomes may not be immediate, and thus, they may be difficult to measure.

A good measurement criterion for health care social media should reflect its technological features and health care context. As an IS artifact, social media needs to be evaluated based on outcomes achieved after user-system interaction. However, given that the context is health care, these outcomes should be wellness. On the basis of this argument, the value of health care social media seems to be the positive outcomes it delivers to the intended recipient after interaction with the application.

#### Utilitarian and Hedonic Value

Effective social media platforms are *sticky*, meaning that users visit them frequently and also spend a fair amount of time on them [50]. Continuing to use a social networking platform indicates that the user must experience some benefits or satisfaction from it [51]. After signing up for social media, the value of the services becomes a factor in their continued usage. Users must receive some perceived value or benefits for using the network, otherwise there would not be any motivation for users to continue using the network after joining [51]. The value users derive from using health care social media could reflect hedonic or utilitarian value.

IS value can be analyzed based on the type of value the IS artifact being studied delivers, that is, whether they provide hedonic or utilitarian value [43]. Hedonic value has to do with a product’s entertainment-related or emotional benefits, whereas utilitarian value has to do with what the product does or what it allows the user to do [52].

The literature reviewed suggests that users usually do not use health care social media for entertainment. Rather, they use the application because they see it as a medium to complete some necessary tasks, such as accessing or sharing health-related information. People that use social media for health-related activities have a clear intent for using the application; thus, they deliberately choose media that will satisfy given needs in terms of knowledge, social interactions, companionship, diversion, or escape [53]. In other words, social media is used in health care because of its functional value. Functional value represents value derived from effective task fulfillment [44].

#### Instrumental and Intrinsic Value

The value of social media in health care settings can be considered either instrumental or intrinsic. Instrumental value is the property that allows something to serve as a means toward getting something else that is valuable [54]. A growing number of the literature [1,13,15] discuss how using social media in health care settings could yield value. Such uses can be said to be instrumentally valuable given that it serves as a means toward getting value. From this perspective, using social media in health care is not valuable in itself but valuable because it leads to other benefits. One way of measuring the instrumental value of social media in health care settings is to evaluate its capacity to develop engaged audiences in health promotion settings [24]. In this example, the use of social media is not valuable in itself, but a means to achieving the desired value which is an engaged audience.

On the contrary, intrinsic value is the property that makes something valuable in itself, as opposed to being a means to something else [54]. From this philosophical point of view, social media can be said to have intrinsic value in health care settings. In health care settings, the intrinsic value of social media can be measured by evaluating its impact on health care [22,55,56].

#### Contextual Value

Value does not automatically arise from the properties of an IS, rather it results from the interaction of a user and a given IS in a particular context [44]. In IS research, technology is always linked with context. An IS such as health care social media can be used in different contexts; therefore, it is important to clarify what the use context is to ascertain its value [31]. To understand the value of an IS, it is essential to identify what is important to its users, that is, the value that users desire [44]. Clarifying the use case of an IS when studying its value yields clearer results [43,57,58]. That said, in health care, stakeholders often have numerous mutually exclusive objectives such as access to services, high quality, cost containment, safety, convenience, patient-centeredness, and satisfaction [30].

Although users’ objectives and contexts of use are relevant to understanding the value of an IS, many IS value research studies have failed to address the impact of contextual factors [58]. The literature exploring this subject as it relates to the use of social media in health care is scarce. However, a few studies [1,13,15] explored why social media is used in health care and identified 9 categories of reasons: public health promotion, organizational promotion, professional networking, professional education, patient care/education, research, peer support, crowdsourcing health initiatives, and harnessing patients’ feedback. This information is important because understanding what users are ultimately trying to accomplish is a prerequisite to defining the appropriate measurement criteria and which measurement tools to use [26].

### Focus of Analysis

IS value research broadly fits into 3 categories based on their objectives: improving design, making IS yield consistent benefits, and creating and deriving value from IS [43]. The first category of IS value research seeks to improve design and implementation by elucidating users’ motivations, the impact of IS on behavior, and how IS creates utility for users. The second type of IS value research investigates how to make expensive IS investments yield effective and consistent benefits; thus, they explore the impact of IS on the employees, processes, customers, and society. The third research strand, among other things, is concerned with how value is created and derived by users. Most literature reviewed for this study falls into the second category, as most explored the impact of social media on health care providers [6], processes [59], patients [60], and the wider society, for example, in public health surveillance [61,62].

Broadly speaking, on the basis of the focus of their analysis, 3 streams of the literature on measuring the value of social media in health care emerged from the review. The first stream described the potential value of social media in health care [6] and the need to measure the outcomes of social media initiatives [15,31]. The second stream focused on how to measure the value of social media used in health care settings based on outcomes resulting from its use [22,55,63,64]. Finally, the third stream presents how data derived from social media can be used for measurements relevant to health care [65-67].

### Period of Analysis

IS value research can also be categorized based on the period of an IS artifact’s life cycle that is the focus of analysis. To this end, there are 2 types of IS value research: preadoption IS value research and postadoption IS value research [43,58,68].

#### Preadoption

Preadoption IS value research helps decision makers to understand which of the available IS options will most likely yield desired results. Going by the literature reviewed in this study, less or no research studies have focused on the preadoption of social media in health care, probably because trying to measure upfront does not yield much [5]. This suggests that many health care providers are adopting and using social media without knowing whether they are choosing the correct option for their context. If that is the case, then more research needs to be conducted in the area of preadoption of health care social media to help decision makers to understand which of the available social media options (eg, general purpose social media vs online health community) will most likely yield desired results.

#### Postadoption

Postadoption IS value research investigates the extent to which IS investments have actually yielded the desired value [43,58,68]. Virtually, all the literature reviewed in this study fits into this category. Most of the available literature examined the value of social media to health care providers that are already using the application. Postadoption value research is important because it yields information that allows health care providers to determine whether they have derived value from their social media initiatives.

### Metric for Measurement

IS value can be appraised either by using monetary measures or by using nonmonetary measures [43]. Although many advocates that metrics that measure monetary value be used to measure the value of social media in health care, however, metrics that measure nonmonetary value are equally relevant given that a substantial part of IS value is nonfinancial or intangible in nature [58]. Furthermore, health care providers are often not profit seeking; thus, the value of the use of social media in health care may not be effectively measured using only monetary metrics.

#### Monetary

When used for organizational promotion, the impact of social media on a health care practice can be determined using metrics that measure return-on-investment (ROI), that is, the gain or loss generated on the application relative to the amount of money invested [2,28]. Similarly, one can also measure the value of social media in health care by examining how social media compares with alternative mediums of professional medical education using cost analysis, that is, by comparing the ratio of benefits over costs [69]. Furthermore, medical literature [30,48,49] proposes that value be measured in health care settings based on outcomes achieved per money spent. This suggests that the value of social media in health care may be measured based on outcomes achieved from using the application per amount of money spent on social media initiatives. However, there is a need to distinguish between performance, which is measured by means of monetary indicators, and its (potentially different) values in terms of the subjective interpretation of (different) stakeholders [58]. In other words, the subjective nonmonetary perceptions of stakeholders are an element of value.

#### Nonmonetary

There are a wide variety of nonmonetary yardsticks that can be used to measure the value of social media in health care. For example, when used for public health promotion, the value of social media in health care can be measured based on its impact [70] and reliability [61]. Furthermore, the value of social media in health care can be analyzed in terms of its ability to capture patient-generated information [62] or based on health outcomes resulting from the use of the application [56,60,71] Other nonmonetary yardsticks for appraising the value of social media in health care include measuring its leveragability to drive health improvement [72,73], appraising the extent to which it increases outreach [74], or evaluating it based on the extent to which it facilitates audience engagement [24].

### Measurement Level

The value of social media in health care can be measured either at the micro or at the aggregate level. Alternatively, the value of social media in health care can be measured at the international, national, industry, organizational, or individual levels.

#### Micro and Aggregate Level

When measuring the value of social media, it is important to measure at different levels to see big picture results and see more granular results [26]. The value of social media can be appraised at the micro or aggregate levels [43].

Analysis of value at the micro level focuses on the *individual* or *organization*, whereas analysis at the aggregate level focuses on the *network* or *society* [43]. For instance, at the individual level, users’ perspectives are paramount when exploring the value of social media use [15,27]. At the organizational level, on the contrary, it is a bit more complicated to establish the value of social media use; thus, a taxonomic approach is recommended to facilitate a better understanding of the phenomenon [32]. Finally, at the societal level, the measurement complexity is more pronounced; thus, it is very difficult to measure the impact of digital artifacts such as social media on the society at large [75].

#### International, National, Industry, Organizational, and Individual Level

IS value research evaluates the worth, desirability, or utility of artifacts at various levels, such as the society, firm, organizational network, and individuals [43]. The value of an IS can be analyzed at the international, national, industry, organizational, and individual level [76]. Thus, the value of social media in health care can be analyzed at the international, national, industry, organizational, and individual level.

At the international level, the value of social media in health care can be viewed in terms of the ability of the application to support the creation of online health communities that allow health care providers from around the world to interact, without the limitation of geographical location [9]. At the national level, the value of social media in health care can be measured by, for example, evaluating the social media performance of hospitals in a particular country [77]. At the industry level, there are different plausible ways to explore the value of social media in health care, for example, by measuring to see whether hospitals’ social media ratings reflect their actual level of competence [66], by measuring to determine the value of social media to the medical profession [6], or by evaluating the potential benefits of using social media to facilitate continuing medical education[69,73]. At the organizational level, the value of social media can be understood in terms of its value in organizations [32], by, among other approaches, measuring the ROI on the application [2]. Finally, at the individual level, the value of social media in health care can be measured by, among other options, exploring the motivations and consequences of its use [27] or by comparing outcomes with the value users desire from the application [15].

### Measurement Approach

When measuring the value of social media in health care, it is important that the right measurement approach is chosen. There are various approaches to measurement that are available for measuring the value of social media in health care. There is the choice of using a qualitative, quantitative, or mixed method approach.

#### Quantitative

The quantitative approach to measurement is the systematic empirical investigation of observable phenomena via statistical, mathematical, or computational techniques [78]. Most relevant theoretical and empirical research papers appear to be concerned with quantifying impact in health care settings. At the basic level, social media impact can be measured by quantifying the number of hits, page views, visits, return visits, unique visitors, cost per unique visitor, time spent, and interaction rate [79]. Some of the other quantitative methods suggested in the literature reviewed include calculating to determine the ROI of social media [2,28], determining the online influence of a health care provider that uses social media by calculating their Klout score [80], and using a cost-benefit analysis to determine the value of social media in health care [69]. Another quantitative metric for measuring value in health care settings is to calculate health outcomes achieved per dollar spent [30,48,49].

#### Qualitative

The qualitative approach to measurement is a systematic method of observation that is used to gather nonnumerical data [78]. This type of research identifies meanings, concepts, definitions, characteristics, metaphors, symbols, and description of things and not to their counts or measures [78]. The qualitative method is particularly relevant to measuring the value of social media in health care because its value may manifest in ways that quantitative metrics alone would be unable to capture. Intangible value created by IS artifacts is as important as other types of IS value [58]; therefore, relying solely on traditional measures such as hit counts or other quantitative methods to measure IS value may yield less accurate results than if some level of qualitative analysis is incorporated in the measurement process [40]. Some of the qualitative methods that have been used to explore the value of social media in health care include sentiment analysis [65], taxonomy [3,15], and analysis based on uses and gratification [53].

#### Mixed Method

An alternative to both quantitative and qualitative methods is the mixed method research approach. Mixed method is a research approach that uses multiple methods—more than one research method or more than one worldview [81]. It may combine quantitative and qualitative research methods in the same research inquiry to develop rich insights into various phenomena of interest that cannot be fully understood using only one research method [81].

The review of relevant literature suggests that mixed method is relevant to investigating the value of social media in health care settings, especially in instances where one method alone may not effectively capture the outcomes resulting from using the application. For instance, using mixed method to research the value of social media in health care allows one to measure the level of audience engagement, determine what resonates with the audience, and changes resulting from behavioral and educational interventions [72]. Some of the ways mixed method has been used in health care social media research include combining social network analysis with content analysis [82], combining content, thematic, and comparative analysis [83], and combining experiment with qualitative analysis [84].

### Measurement Scale

Beyond choosing whether to adopt a qualitative or quantitative approach to measurement, those seeking to measure the value of social media in health care would need to choose the specific scale of measurement to adopt. According to Stevens [36], all types of measurement can fit into at least one of the following measurement typologies: nominal, ordinal, interval, and ratio.

#### Nominal Scale

The nominal scale is an unrestricted assignment of numerals, words, or letters to events or objects simply as labels or unique identifiers to indicate distinct types [36]. The nominal scale is regarded as the most basic form of measurement and is used to categorize and analyze data in many disciplines. When measuring the value of an IS, the scale level of a value item does not necessarily have to be *cardinal* when it is difficult or even impossible to find reliable numerical data [58]; hence, an alternative yardstick such as the nominal scale could be used.

The literature review revealed that the nominal scale is relevant to appraising social media in health care, as it is used for categorization. For instance, health care social media can be grouped into 2 broad categories, namely, general purpose Web-based social networks and online health communities [3]. general purpose Web-based social networks are websites such as Facebook, Twitter, Instagram, and YouTube that enable mass collaboration. Although these platforms are not designed specifically for patients and health care–related communication, their features and functionalities make them suitable for health-related communication [3]. online health communities, on the contrary, are social media websites such as MedHelp and PatientsLikeMe, which are specifically designed for health-related communication [3]. Going further, the use context of social media in health care can be categorized into 4 broad categories based on participants: P2P, P2C, C2C, and C2P [3] At the granular level, the uses of social media in health care can be grouped into 9 contexts of use: professional networking, harnessing patients’ feedback, public health promotion, professional education, patient education, organizational promotion, crowdfunding health initiatives, research, and peer support [15].

The advantage of using a nominal scale is that it can help with classification of types of social media used in health care and the context in which they are used. It can be used to determine mode; for example, the most used type of social media (eg, general purpose or online health communities or Facebook, Twitter, and others) in health care. From reviewing relevant literature, it is apparent that general purpose social media are some of the most used type of social media in health care. The general-purpose social media most referred to in the literature are Facebook [56,66,83] and Twitter [24,65,82].

#### Ordinal Scale

Ordinal level of measurement is the second of the 4 measurement scales. It is used to measure a categorical, statistical data type where the variables have natural, ordered categories but the distance between the categories is not known [36]. Ordinal scales are relevant to measuring health care social media in situations where there is a need to compare phenomena. For example, anecdotal evidence suggests that some health care providers use social media more effectively than others [31]. Going by that assertion, health care providers that use social media can be ranked based on how effectively they use the application: highly effective user, effective user, and ineffective user. This kind of ranking allows data regarding health care social media to be sorted; however, it does not allow the relative degree of difference between them to be determined. On the basis of the ranking, one can tell that one user is more effective than the other at using social media; however, the extent to which a highly effective user is better than an effective user cannot be described. Similarly, patients’ perceived quality of care could be measured based on Twitter data [65]. Speculating based on that assertion, patients’ perceived quality of care can be ranked based on quality level, for example, high quality, medium quality, and low quality. The median can be determined in this way, but the average cannot. The rankings indicate the patient’s perceived quality of care received but does not provide sufficient information to determine the exact difference in quality between the various categories.

#### Interval Scale

The interval scale is a *quantitative* measurement scale in which the order and the exact differences between the values are known, but that does not imply the existence of a *true* zero point [36]. The interval scale allows for the degree of difference between items to be determined, but not the ratio between them, for instance, date when measured from an arbitrary epoch such as anno Domini (AD) or before Christ (BC. Using these scales, it may be misleading to say that one value is twice or some other proportion greater than another.

Interval scales are relevant for measuring social media in health care. Any measurement criteria that include time or date, technically include an interval scale, since hours and days are all interval measurements. For instance, interval scales can be used to identify optimal times for engagement and to tailor strategy so the right content is posted at the right time (when the audience is most active) and the best days of the week. Through Facebook insights, one can view their followers’ daily activity over the past week and can go further and narrow it down to individual days to see how engagement shifts by the hour. By being able to see how audience engagement with their social media changes over a designated period, one can determine which periods are best for bringing in new fans. Using an interval scale to measure social media, one could find that evenings are better than mornings or that weekends are better than weekdays when it comes to audience engagement. That would allow for the degree of difference between these periods to be known but not the ratio between them, given that terms such as *weekdays*, *weekends*, *mornings*, and *evenings* are ambiguous.

#### Ratio Scale

The ratio scale is a quantitative variable measurement scale that allows comparisons to be made between intervals or differences. Ratio scales are used to estimate *the ratio between a magnitude of a continuous quantity and a unit magnitude of the same kind* [85]. Unlike the interval scale, the ratio scale possesses a nonarbitrary zero value; thus, most measurements in the physical sciences fall under this category.

Ratio scales are widely used in social media measurement, and most social media metrics are examples of the ratio scale. For instance, ratio scales allow social media managers to measure the number of page views, visits, return visits, unique visitors, cost per unique visitor, time spent, and interaction rate [79]. Furthermore, when measuring social media performance, ratio scales can be used to calculate the impressions-to-interactions ratio [28]. Ratio scales can be used to determine how much (magnitude and amount) of something. In health care settings, for instance, the magnitude of a research recruiter’s influence can be determined by calculating their Klout score [80]. Influence in this context refers to a measure of a user’s ability to drive action from their posts or social interactions. In financial terms, ratio scales can be used to determine the amount of financial benefit a health care provider derives from using social media. For instance, a health care provider can use the ratio scale to calculate the ROI of social media to their practice [2,28].

Ratio scales can also be used to determine how many (count) of something. For example, ratio scales can be used to determine how many health care providers use social media or a particular type of social media [86,87].

## Discussion

### Summary of Findings

The following taxonomic categories were created based on the literature reviewed: type of article, type of value, period of analysis, measurement metric, measurement level, measurement approach, and measurement scale. From the review of relevant literature, we found the following:

1. Articles on social media value can be derived from IS/information technology (IT), business, medical, or medical informatics literature.
2. Value to measure when appraising health care social media could be IS/IT value or health value, utilitarian or hedonic value, instrumental or intrinsic value, or contextual value.
3. The value of social media in health care can be analyzed pre–social media adoption or post–social media adoption.
4. Metrics used to evaluate the value of social media in health care can measure for either monetary or nonmonetary value.
5. In health care settings, the value of social media can be measured at the micro or aggregate level. Alternatively, it can be measured at the international, national, industry, organizational, and individual levels.
6. The measurement approaches for measuring social media value in health care settings can be quantitative, qualitative, or mixed method in nature.
7. One or more of the following measurement criteria is used in the measurement of health care social media: nominal scale, ordinal scale, interval scale, and ratio scale.

### Significance of Study

Research aimed at developing measurement criteria for health care social media is important for both theory and practice. From a theoretical standpoint, investigating the measurement criteria of social media supports the accumulation of knowledge in this emerging domain [40]. From a practice point of view, identifying useful measures of social media outcomes enables managers to evaluate the consequences of social media initiatives* *vis-à-vis the objectives, enabling them to manage social media strategies from positions that are less reactionary and more grounded in established knowledge or theory [40].

Although there are many papers that describe plausible yardsticks for measuring the value of social media in health care settings, this paper is the first to provide a taxonomic review that covers the types of literature on the subject, the period of analysis, and the focus of analysis. It also covers types of value, metrics for measurement, levels of measurement, approaches to measurement, and scales of measurement.

This study aimed to resolve the dilemma regarding what to measure and how to measure by reviewing and synthesizing relevant literature on the subject. This review paper constructively informs stakeholders about what has been learned, what patterns have emerged from the literature, and how research builds upon previous findings [38]. The findings of this study will help health care managers to ascertain the role of social media in health care and to design social media strategies that can yield tangible results.

This study lays the foundation for the development of a framework to help health care providers measure the outcomes of their social media initiatives by explaining current metrics, yardsticks, and tools for measuring its effectiveness. This is critical because without metrics derived from a theoretical understanding of the underlying processes and objectives, the suitability of the framework may be contestable [40].

### Limitations

This work aimed to analyze the literature related to social media value to solicit a taxonomy of ways in which value has been conceptualized. The literature reviewed was not intended to be a systematic review, and it is possible that a wider review may have identified articles that suggest additional concepts for the taxonomy.

### Conclusions

This review confirmed a diversity of criteria for measuring social media in health care settings. The most important findings being that users are not sure what types of value to look for, what scale of measurement to deploy, what type of measurement to conduct, when to measure, what metrics to use, and whether to measure at the micro or macro level.

At this time, there is no definitive literature or comprehensive set of methods for measuring the short-, medium-, and long-term impacts of social media on health care quality and safety [72]. Addressing these gaps through more (robust) research is likely to uncover simplified criteria for measuring all types of social media and all contexts of use. Furthermore, as social media is only a tool among several digital technologies that are used in health care, it could be difficult to tease out the specific contributions of social media. However, this seeming challenge of measurement should not be perceived as an obstacle but rather an opportunity to design and develop suitable yardsticks for measuring the contributions of social media in health care settings.

As social media continues to permeate health care, having the measures available and in place to monitor and evaluate outcomes over time will help ensure the effective use of social media in health care improvement.

### Recommendations

At the start of a health care social media initiative, a comprehensive measurement approach should be put in place. This will ensure that attention is focused on the objectives and enable the verifiability of outcomes [26]. Users need to clearly define what success will look like [26]. Articulating the objective will help define the appropriate metric to adopt and which measurement tools to use [26]. A starting point could be to understand what others are doing with social media and compare strategies [5].

To determine an appropriate measurement criterion for health care social media, there is a need to define the objectives of the social media initiative, the target audience, and the outcome desired from a social media campaign [72]. Thus, when choosing a measurement framework, it is important to define the metrics to capture in terms of the use cases, importance, and specific objectives [26].

We recommend that this review be used as a starting point to further elucidate and create appropriate measurement criteria for health care social media. Relevant steps in this direction would include the identification of value items with which the respective value can be measured and the identification and development of methodologies that allow measurement [58].

## Abbreviations

C2C: consumer to consumer
C2P: consumer to professional
IS: information system
IT: information technology
P2C: professional to consumer
P2P: professional to professional
ROI: return-on-investment
